# Clozapine Induced Disturbances in Hepatic Glucose Metabolism: The Potential Role of PGRMC1 Signaling

**DOI:** 10.3389/fendo.2021.727371

**Published:** 2021-12-14

**Authors:** Ting Cao, Qian Chen, BiKui Zhang, XiangXin Wu, CuiRong Zeng, ShuangYang Zhang, HuaLin Cai

**Affiliations:** ^1^ Department of Pharmacy, Second Xiangya Hospital, Central South University, Changsha, China; ^2^ Institute of Clinical Pharmacy, Second Xiangya Hospital, Central South University, Changsha, China

**Keywords:** clozapine, hepatic glucose disturbances, PGRMC1, gluconeogenesis, glycogenesis

## Abstract

Newly emerging evidence has implicated that progesterone receptor component 1 (PGRMC1) plays a novel role not only in the lipid disturbance induced by atypical antipsychotic drugs (AAPD) but also in the deterioration of glucose homoeostasis induced by clozapine (CLZ) treatment. The present study aimed to investigate the role of PGRMC1 signaling on hepatic gluconeogenesis and glycogenesis in male rats following CLZ treatment (20 mg/kg daily for 4 weeks). Recombinant adeno-associated viruses (AAV) were constructed for the knockdown or overexpression of hepatic PGRMC1. Meanwhile, AG205, the specific inhibitor of PGRMC1 was also used for functional validation of PGRMC1. Hepatic protein expressions were measured by western blotting. Meanwhile, plasma glucose, insulin and glucagon, HbA1c and hepatic glycogen were also determined by assay kits. Additionally, concentrations of progesterone (PROG) in plasma, liver and adrenal gland were measured by a liquid chromatography-tandem mass spectrometry (LC-MS/MS) method. Our study demonstrated that CLZ promoted the process of gluconeogenesis and repressed glycogenesis, respectively mediated by PI3K-Akt-FOXO1 and GSK3β signaling *via* inhibition of PGRMC1-EGFR/GLP1R in rat liver, along with an increase in fasting blood glucose, HbA1c levels and a decrease in insulin and hepatic glycogen levels. Furthermore, through PGRMC1-EGFR/GLP1R-PI3K-Akt pathway, knockdown or inhibition (by AG205) of PGRMC1 mimics, whereas its overexpression moderately alleviates CLZ-induced glucose disturbances. Potentially, the PGRMC1 target may be regarded as a novel therapeutic strategy for AAPD-induced hepatic glucose metabolism disorder.

## Introduction

It is well recognized that abnormal glucose and lipid metabolism is a high-risk factor of cardiovascular and cerebrovascular events, contributing to the premature death of schizophrenic patients from physical diseases greatly (1). CLZ, the prototype of AAPD, has been widely described in treatment-resistant schizophrenia with superior efficacy. However, subjects treated with CLZ show a high risk of developing different metabolic side effects, such as body weight gain, glucose homeostasis disturbance, abnormal insulin secretion and diabetes as compared to the general population (2). Preclinical evidence has indicated that both acute and chronic treatment with AAPDs disturbed glucose homeostasis in rats (3). Furthermore, the discontinuation of antipsychotic drugs leads to the restoration of normal glucose control in patients (4). Therefore, it is possible that AAPDs directly regulate glucose homeostasis. Despite the number of clinical reports continues to grow, the molecular mechanism by which CLZ could cause glucose dysregulation is still largely unclear. It has been found that CLZ could accumulate in liver tissue, reaching certain levels up to 10 μmol or higher, which are several folds higher than plasma concentrations (5). Chronic CLZ treatment has been associated with an increased risk for disruption of glucose homeostasis, leading to hyperglycemia and insulin resistance and/or type 2 diabetes. As reported previously (6), the possible mechanisms underlying hyperglycemia are due to impairment of insulin action (7) and insulin resistance, or may be due to certain defects of metabolic pathways leading to either glycogenesis decrease or accelerated glycogenolysis (2, 8–10).

PGRMC1, as a type of adaptor protein, is directly involved in cell signaling by trafficking and interacting other transmembrane receptors to the plasma membrane. A recent translational study demonstrated decreased levels of PGRMC1 protein and RNA in patients with insulin resistance, suggesting a role of PGRMC1 in insulin signaling (11, 12). PGRMC1 has also been found to modulate glucose-induced insulin stimulation and to interact with the activated the liganded glucagon-like peptide-1 receptor (GLP1R), contributing to glucose homoeostasis in beta cells (13, 14). From a clinical standpoint, the disruption of glucose homeostasis generally accompanies with lipid disturbance among the adverse metabolic effects induced by AAPD. In our previous study (15), it has been demonstrated that the PGRMC1/INSIG-2 signaling exerted regulatory effect in AAPD-induced lipid disturbances in liver. Hence, we reckon that the PGRMC1 signaling is potentially involved in the glucose disturbances induced by clozapine treatment as well.

The liver plays a central role in the maintenance of blood glucose by balancing new synthesis (gluconeogenesis) and glycogen synthesis (16). Under physiological conditions, insulin stimulates 3-phosphoinositide-dependent kinase-1 (PI3K) and the cascade of signaling event leading to the activation of protein kinase B (Akt), which is considered to repress hepatic glucose production mainly through two key mechanisms: first, inhibition of gluconeogenic enzymes by phosphorylation and nuclear exclusion of the fork head box protein 1 (FOXO1) (17) and second, activation of glycogen synthase by phosphorylation and inactivation of glycogen synthase kinase-3β (GSK3β) (18).

Intriguingly, new evidence provided support for the coprecipitation and colocalization between PGRMC1 and epidermal growth factor receptor (EGFR) in cytoplasmic vesicles in cells (19). Further, PGRMC1 also serves as a novel component of GLP1R complex (13). Evidence has revealed the presence of GLP1R in rat liver, involved in the regulation of glucose balance in the body (20). EGFR and GLP1R are both well-established upstream regulators of the PI3K-Akt pathway (21–23). Therefore, it was likely that PGRMC1 dually regulates the PI3K-Akt pathway *via* the interaction with GLP1R and EGFR.

The present study aims to evaluate the effects of 4-week CLZ treatment on glucose homeostasis and hormone secretion in male rats. We also examined whether the PGRMC1 signaling is involved in the CLZ-induced disruption of glucose homeostasis. On the basis of above, the dual regulatory effects on knockdown and overexpression of PGRMC1 on EGFR/GLP1R-PI3K-Akt pathway were further validated in CLZ-induced hepatic glucose dysregulation.

## Material and Methods

### Animals

To avoid possible influence of cyclic, systemic PROG fluctuation caused by menstruation cycle (15), only male Sprague–Dawley rats were adopted in our study. Rats weighing between 180 and 220 g (approximately 5 weeks old) were purchased from Hunan Slack Jingda Experimental Animal Co., Ltd (Hunan, China). In experiment 1, 9 rats (n=3/group) were used to assess the regulatory effects of AAV on PGRMC1 expression in basal conditions. In experiment 2, a total of 45 rats (n=9/group) were used for exploring the potential mechanism underneath CLZ-induced glucose disturbances. All rats were housed under conditions with a light-dark cycle (12 h/12 h) and room temperature (24-25°C) with free access to food and water. The animal research protocol was approved by the local Ethics Committee of the Second Xiangya Hospital of Central South University (Approval No. 2020008). All efforts were made to minimize the animal suffering and the number of animals used.

### Chemicals and Reagents

CLZ was purchased from Wuhan Yuancheng Co-Creation Technology Co., Ltd. (Wuhan, China). AG205 (purity≥97.5%) was synthesized by Jining Drug Research and Development Center. CLZ (4 mg/ml, 20 mg/kg/day) and AG205 (4.24 mg/ml, 21.2 mg/kg/day) were initially dissolved in 5% (v/v) ethanol and then further diluted in 0.9% saline water containing 22.5% 2-hydroxypropyl β–cyclodextrin to obtain the final concentration. All the solutions were injected intraperitoneally. The CLZ dose used in our experiments was selected on the basis of our previous research (15), which was converted from clinically prescribed dosages. Due to lack of evidence about the dose usages of AG205 in animal models, we calculated the AG205 dose converted from CLZ based on their molecular docking score binding to PGRMC1 (Detailed information of the calculation process is provided in **Supplementary Tables 1–3**). Additionally, the inhibitory effect of AG205 on hepatic PGRMC1 expression in basal conditions has been validated as seen in **Supplementary Figure 1**.

### Construction Of Recombinant Adeno-Associated Viruses

The AAV tools targeted at PGRMC1 as well as the control vectors were designed and provided by Hanbio Biotechnology Co., Ltd. (Shanghai, China). Specifically, there are two types of AAV used in our study including AAV/BBB 2.0-CMV-r-PGRMC1-3xflag-GFP (PGRMC1-OE, 1.1×10^12^ vg/ml) for PGRMC1 overexpression and AAV/BBB 2.0-r-PGRMC1 shRNA3-GFP (PGRMC1-KD, 1.1×10^12^ vg/ml) for PGRMC1 knockdown. The shRNA sequence targeting rat PGRMC1 is AATTCGATTGTGTACTCGGATGATGAAGAATTCAAGAGATTCTT-CATCATCCGAGTACACAATCTTTTTTG (Top strand); GATCCAAAAAAGATTG-TGTACTCGGATGATGAAGAATCTCTTGAATTCTTCATCATCCGAGTACACAATCG (Bottom strand). Detailed information about the genetic tools has been provided in supplementary material.

### Experimental Design

**Figure 1:**
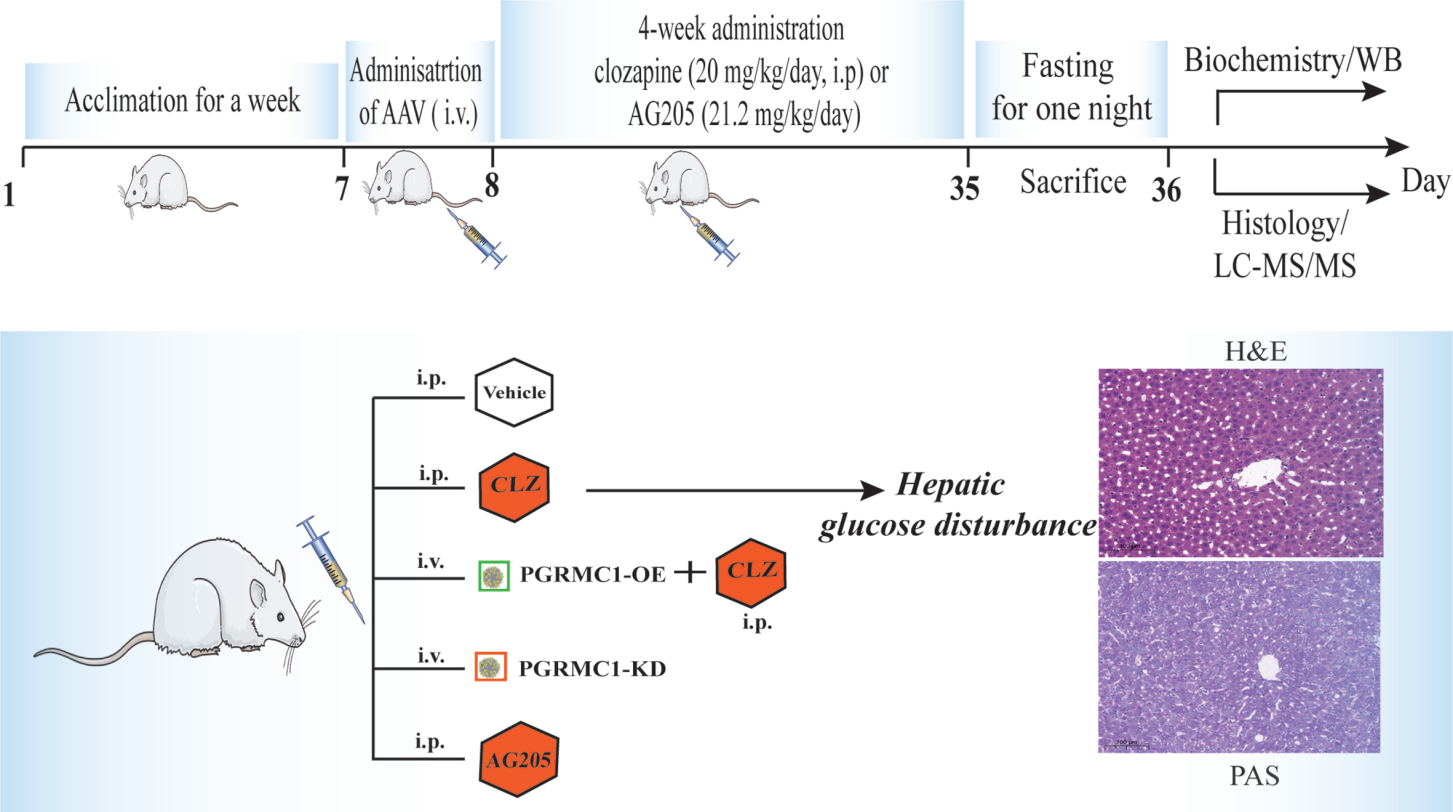
A diagram of the time course illustrating when each procedure took place.

The regulatory effects of AAV which are designed to upregulate and downregulate the hepatic PGRMC1 expression were verified in the first batch of rats with a division into three groups (*n*=3/group): the vehicle group (NC), down-regulated AAV group (PGRMC1-KD) and overexpression AAV group (PGRMC1-OE). All kinds of AAV were injected *via* tail vein only once with a volume at 300 μl. In 28 days, anatomy was exerted to observe the GFP immunofluorescence in liver sections and the rat liver tissues were collected for western blot analysis. The second batch of rats were divided into five groups (*n*=9/group): the vehicle group (NC), down-regulated AAV group (PGRMC1-KD), and blank AAV + CLZ group (CLZ, 20 mg/kg/day), CLZ + overexpression AAV group (CLZ+PGRMC1-OE), AG205 group (**Figure 1**). All rats were acclimatized for one week before experimentation. All kinds of AAV were injected *via* tail vein for only once, while blank solvent, clozapine and AG205 were administered by intraperitoneal injection continuously for 28 days. All rats were fasted for 12 h before sacrifice. After being anaesthetized with 2% pentobarbital sodium solution (0.2 ml/100 g), the rat liver tissue samples were collected and frozen immediately in liquid nitrogen. Whole blood was collected from the truncal vessel in EDTA anticoagulation vacuum tubes. The plasma was obtained by centrifugation of blood at 4°C, 3000 rpm for 15 minutes and stored at −80°C before analysis (**Figure 1**).

### Histological Examination and Biochemical Assays

The collected liver tissues were soaked in fixative overnight. Then the tissues were placed in a 30% PBS solution for 2–3 days and sectioned at freezing stage. GFP was immuno-labeled using rabbit anti-GFP primary antibody (Cat#: 50430-2-AP, Proteintech, Wuhan) and Fluorescein (FITC)-conjugated Affinipure Donkey Anti-Goat IgG (H+L) secondary antibody (SA00003-3). Liver samples were fixed in 4% paraformaldehyde overnight at 4°C, embedded in paraffin and sectioned at 4-μm thickness. Tissue sections were stained with hematoxylin and eosin (H&E). Then, the histopathological examination was performed under light microscopy. Periodic acid–Schiff (PAS) staining was used to visualize the glycogen deposits in liver tissues. Liver samples were soaked in 4% paraformaldehyde solution and then mounted in paraffin and sectioned. Sections were oxidized in periodic acid for 20 min at room temperature and rinsed for three times in deionized water. The liver sections were counterstained with haematoxylin and eosin for 1 min, rinsed in deionized water and observed for the red-staining parts as the presence of glycogen using a light microscope.

Plasma glucose and hepatic glycogen levels were tested using Biochemical Assay Kits (Glucose, Cat#: BC2495; Glycogen, Cat#: BC0345; all obtained from Solarbio LIFE SCIENCES, China) following the manufacturer’s instructions. Insulin, HbA1c and glucagon levels were measured by immunoassays using commercial ELISA Kits (Insulin, Cat#: CSB-E05070r; HbA1c, Cat#: CSB-E08140r; Glucagon, Cat#: CSB-E12800r; all purchased from CUSABIO, China).

### Determination of PROG Levels in Liver, Plasma, and Adrenal Gland

Aliquots of 0.1 g of liver/adrenal gland tissue were homogenized and then the tissue homogenate or plasma (300 uL) was transferred and mixed with 1500 μL of methyl tert-butyl ether/methanol (1:1, v/v) extraction solvent, vortexed for 3 min and centrifuged at 20627*g* for 10 min at 4°C. Then the resulting supernatant was evaporated to dryness by a centrifugal vacuum concentrator at 4°C. The residue was next resuspended with 100 μL of 2-propanol/acetonitrile/water (21:9:70, v/v/v) solvent mixture. Finally, the concentrations of PROG in the tissues and plasma were measured by an LC-MS/MS method as previously reported (24, 25). The lower limit of quantifications (LLOQs) of PROG was 0.05 ng/ml for plasma, and were 0.15 ng/g for tissues, respectively. The coefficient of variance (CV) in PROG varied between 2.4% and 9.6% for intra-assay, while for inter-assay the CV in PROG ranged from 3.9% to 7.1%.

### Western Blotting

Proteins from liver tissues were extracted and quantified using a BCA kit as described previously (15, 24). Approximately 20 μg of protein was loaded, electrophoresed, blotted and then incubated with primary antibodies against PGRMC1, EGFR, GLP1R, PI3K p85, Akt, phospho-Akt (Ser^473^), GSK-3β, FOXO1, β-actin, PCNA (Proteintech Group, Wuhan, China), phospho-EGFR, phospho-GSK-3β (Affinity) overnight at 4°C. The membrane was then incubated with appropriate secondary horseradish peroxidase-conjugated antibodies (Boster, Wuhan, China) at room temperature for 1 h and developed color rendering by the ECL kit. The film signal was digitally scanned and then quantified using ImageJ software (National Institutes of Health, Bethesda, MD, USA).

### Statistical Analysis

The data are presented as the mean ± SD and were analyzed using GraphPad Prism 8.0 (GraphPad Software, San Diego, CA, USA). For parameters including weight gain, food intake and feeding efficiency, due to the effects of treatment and time, two-way repeated ANOVA was selected for data analysis. Owing to small samples and non-normally distributed variance, nonparametric Kruskal–Wallis one-way analysis of variance was used for data analysis followed by pairwise multiple comparisons. Statistical significance was considered at *p*<0.05.

## Results

### Alternations of Body Weight Gain, Cumulative Food Intake, and Feeding Efficiency After CLZ Treatment

**Figure 2:**
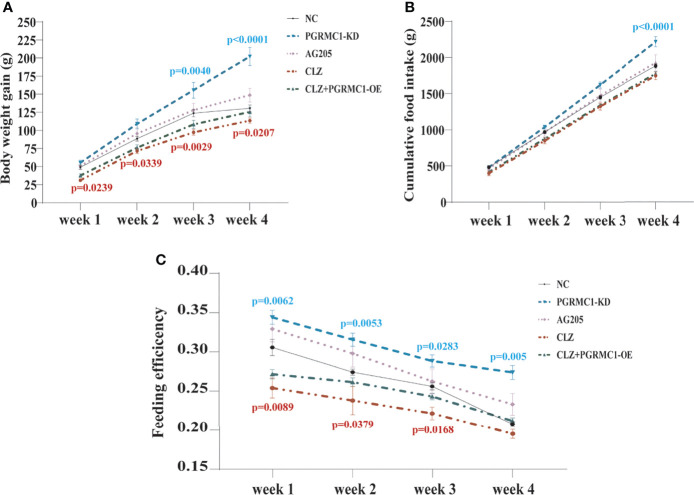
The effect of CLZ on BWG, CFI and feeding efficiency. **(A)** BWG, Two-way repeated ANOVA (Treatment×Time as repeated measures) showed significant main effects of Weeks (*F*_3, 160_ = 238.4, *p* < 0.0001), Treatment (*F*_4, 160_ = 40.79, *p* < 0.0001) with a significant interaction between the two factors (*F*_12, 160_ = 3.604, *p* < 0.0001) on BWG; **(B)** CFI, Two-way repeated ANOVA also revealed that significant main effects of Weeks (*F*_3, 40_ = 929.9, *p* < 0.0001), Treatment (*F*_4, 40_ = 20.26, *p* < 0.0001) with a significant interaction between the two factors (*F*_12, 40_ = 0.0295, *p* < 0.0001) on CFI; **(C)** feeding efficiency, Two-way repeated ANOVA (Treatment×Time as repeated measures) showed significant main effects of Weeks (*F*_3, 40_ = 49.65, *p* < 0.0001), Treatment (*F*_4, 40_ = 33.98, *p* < 0.0001) with no interaction between the two factors (*F*_12, 40_ = 0.8380, *p*=0.6123) on feeding efficiency.

In order to evaluate the effects of 4-week CLZ treatment on glucose homeostasis, we analyzed the body weight gain (BWG), cumulative food intake (CFI) and feeding efficiency of rats. As shown in **Figure 2A**, compared with NC group, a significant attenuation in weight gain in CLZ group was observed from Week 1-4. However, no changes in CFI were observed in the CLZ-treated group, compared to NC group (**Figure 2B**). In order to better reflect the relationship between BWG and CFI, feeding efficiency (grams of weight gained/grams of food consumed) was used as a combined indictor for analysis. Notably, feeding efficiency was significantly decreased in animals treated with CLZ in week 1, 2 and 3 respectively (**Figure 2C**).

### Effects of CLZ on Biochemical Parameters Related With Glucose Metabolism

**Figure 3:**
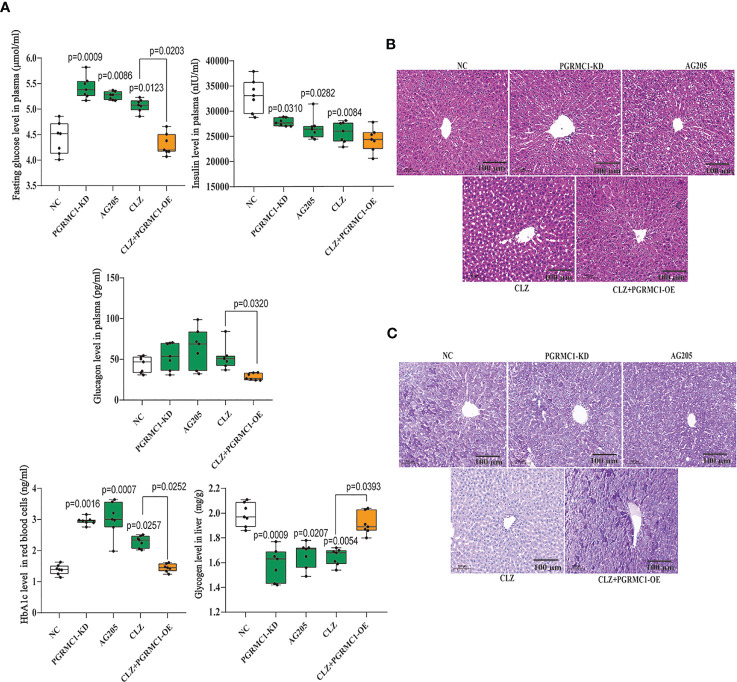
Chronic effects of CLZ on glucose metabolism. **(A)** Biochemical parameters including fasting plasma glucose (*H* = 28.96, *p* < 0.0001), HbA1c (*H* = 28.21, *p* < 0.0001), insulin (*H* = 21.25, *p* = 0.0003), hepatic glycogen content (*H* = 25.56, *p* < 0.0001) and glucagon levels (*H* = 16.08, *p* = 0.0029). **(B)** H&E staining. **(C)** Representative images of PAS staining of liver sections, showing that the decreased glycogen in CLZ-treated rat was prevented by PGRMC1-OE. Red: glycogen; purple: nuclei of liver cells.

In addition, we also measured relevant biochemical parameters related with glucose metabolism. As shown in **Figure 3A**, there were significant differences in fasting plasma glucose (*H*=28.96, *p*<0.0001), HbA1c (*H*=28.21, *p*<0.0001), insulin (*H*=21.25, *p*=0.0003), glucagon (*H*=16.08, *p*=0.0029) and hepatic glycogen (*H*=25.56, *p*<0.0001) among groups. Notably, CLZ-treated rats exhibited increased fasting plasma glucose and HbA1c levels, while reduced the insulin level and hepatic glycogen content, compared with NC group. Although there was an elevated tendency of glucagon levels in CLZ group, the differences did not approach significance.

**Figures 3B, C** showed representative images of randomly selected liver sections stained with Periodic Acid-Schiff stain (PAS) and H&E. The images counterstained with H&E (**Figure 3B**) showed that there were notable histological changes in CLZ treatment group compared with NC group. As expected, PAS analysis (**Figure 3C**) showed that the decreased glycogen distribution in CLZ-treated rat in comparison with NC group, which was restored by PGRMC1-OE.

### PROG Concentrations in Plasma, Liver, and Adrenal Gland

PROG has the strongest binding affinity with PGRMC1 among all hormones (26). To better understand the role of PGRMC1 in CLZ-induced glucose disturbances, the levels of PROG in plasma, liver and adrenal gland were measured respectively. In plasma (*H*=12.53, *p*=0.0138) (**Figure 5D**), we observed increased concentrations of PROG follow CLZ administration. In liver (*H*=21.73, *p*=0.0002) (**Figure 5E**), both CLZ and AG205 markedly increased the levels of PROG. In adrenal gland (*H*=20.15, *p*=0.0005) (**Figure 5F**), treatments with PGRMC1-KD, AG205 and CLZ drastically increased the concentrations of PROG. Despite some parameters did not attain to statistical significance, the PGRMC1-OE displayed opposite effects on the concentrations of PROG in comparison with CLZ group. Specifically, the alternations in hepatic PROG were significantly reversed in PGRMC1-OE group.

### Regulatory Effects of CLZ on PI3K-Akt-FOXO1/GSK3β Pathway in Hepatic Gluconeogenesis and Glycogenesis

**Figure 4:**
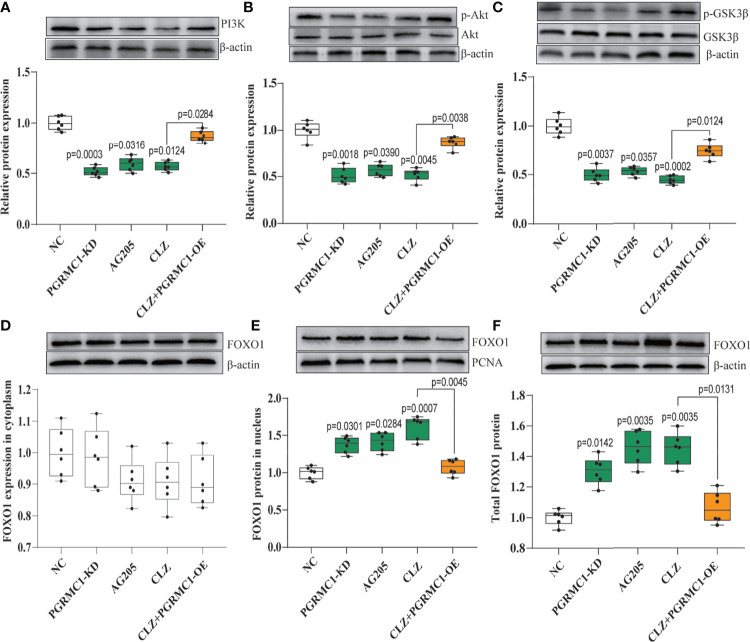
The regulatory effect of CLZ on PI3K-Akt-FOXO1/GSK3β signaling. **(A)** PI3K (*H* = 23.66, *p* < 0.0001); **(B)** the ratio of p-Akt/Akt (*H* = 22.42, *p* = 0.0002); **(C)** the ratio of p-GSK3β/GSK3β (*H* = 24.64, *p* < 0.0001) **(D)** FOXO1 in cytoplasm (*H* = 6.519, *p* = 0.1636); **(E)** FOXO1 in nucleus (*H* = 23.13, *p* < 0.0001); **(F)** total FOXO1 (*H* = 23.30. *p* < 0.0001).

We also examined whether the downstream PI3K-Akt- FOXO1/GSK3β signaling are involved in the clozapine-induced disruption of glucose homeostasis. We found that PI3K (*H*=23.66, *p*<0.0001) and the ratio of p-Akt/Akt (*H*=22.42, *p*=0.0002) was down-regulated in CLZ-treated rats in comparison with NC group (**Figures 4A, B**). Moreover, the nuclear (*H*=23.13, *p*<0.0001) and total FOXO1 (*H*=23.30. *p*<0.0001) were upregulated in CLZ-treated rats (**Figure 4E**). These results indicated that CLZ increased the expression of hepatic FOXO1 while reduced GSK3β phosphorylation (*H*=24.64, *p*<0.0001) *via* inhibition of the PI3K-Akt signaling, thereby activating hepatic gluconeogenesis and inhibiting glycogenesis.

### Effects of CLZ on Hepatic PGRMC1-EGFR/GLP1R Pathway

**Figure 5:**
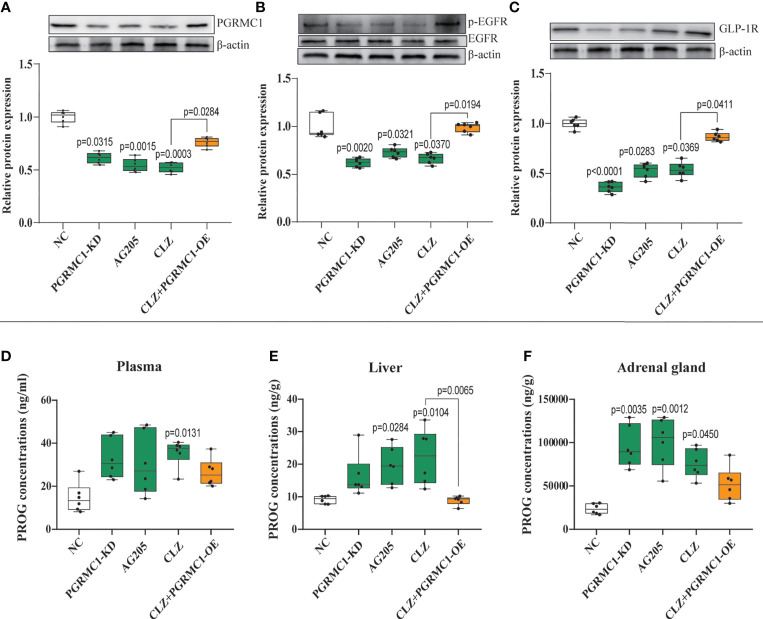
Chronic effects of CLZ on hepatic PGRMC1-EGFR/GLP1R pathway and concentrations of PROG in plasma, liver and adrenal gland. **(A)** PGRMC1 **(B)** the ratio of p-EGFR/EGFR **(C)** GLP1R **(D)** PROG concentrations (ng/ml) in plasma (*H* = 12.53, *p* = 0.0138); **(E)** PROG concentrations (ng/g) in liver (*H* = 21.73, *p* = 0.0002); **(F)** PROG concentrations (ng/g) in adrenal gland (*H* = 20.15, *p* = 0.0005).

To explore the potential modulatory effects of CLZ on the upstream PGRMC1-EGFR/GLP1R signaling pathway, the protein expression of the three key factors (PGRMC1, EGFR, GLP1R) and their phosphorylated forms (p-EGFR, p-Akt) in the liver was compared among groups. CLZ treatment significantly downregulated the protein expression of the three key factors (PGRMC1: *H*=24.71, *p*<0.0001; GLP1R: *H*=26.02, *p*<0.0001; p-EGFR/EGFR: *H*=24.15, *p*<0.0001) compared to NC group (**Figure 5**).

### Effects of Targeting at PGRMC1 on Hepatic Glucose Metabolism

To confirm the role of PGRMC1 in CLZ-induced glucose dysregulation, we utilized the tool of the adeno-associated virus (AAV)-ShRNA-PGRMC1 and (AAV)-CMV-PGRMC1 to knockdown or upregulate hepatic PGRMC1 expression. Moreover, the specific inhibitor of PGRMC1, AG205 was also used for functional validation.

Contrast with CLZ group, a significant increase in BWG was seen in the PGRMC1-KD group in Week 1-4 along with an increase in feeding efficiency in PGMRC1-KD group from week 1-4 compared to NC group (**Figure 2C**). Identical with CLZ group, the PGRMC1-KD and AG205 administration both significantly increased fasting plasma glucose and HbA1c levels, while reduced the insulin level and hepatic glycogen content, compared with NC group (**Figure 3**). Non-significant elevation of glucagon was observed in PGRMC1-KD and AG205 groups. As expected, the PGRMC1-OE markedly reversed the alternations in fasting plasma glucose, glucagon, HbA1c, and hepatic glycogen levels induced by CLZ. These results suggest that PGRMC1 partly contributes to the CLZ-induced abnormal feeding efficiency. Furthermore, the synchronous expression of proteins involved in the PGRMC1 signaling and the downstream PI3K-Akt-FOXO1/GSK3β were also occurred in PGRMC1-KD and AG205 groups. As expected, the PGRMC1-OE ameliorated the modulatory effects of CLZ in protein expression of PGRMC1 pathway. Both PGRMC1-KD and AG205 administration significantly suppressed the protein expressions in PGRMC1-EGFR/GLP1R pathway (PGRMC1: *H*=24.71, *p*<0.0001; GLP1R: *H*=26.02, *p*<0.0001; p-EGFR/EGFR: *H*=24.15, *p*<0.0001) compared to NC group (**Figure 5**). We also found that PI3K (*H*=23.66, *p*<0.0001) and the ratio of p-Akt/Akt (*H*=22.42, *p*=0.0002) was down-regulated in PGRMC1-KD, AG205 in comparison with NC group, but PGRMC1-OE restored the CLZ-induced downregulation of PI3K and p-Akt/Akt (**Figures 4A, B**). In addition, the nuclear (*H*=23.13, *p*<0.0001) and total FOXO1 (*H*=23.30. *p*<0.0001) were upregulated in PGRMC1-KD and AG205, which were eliminated by PGRMC1-OE (**Figures 4E, F**).

## Discussion

**Figure 6:**
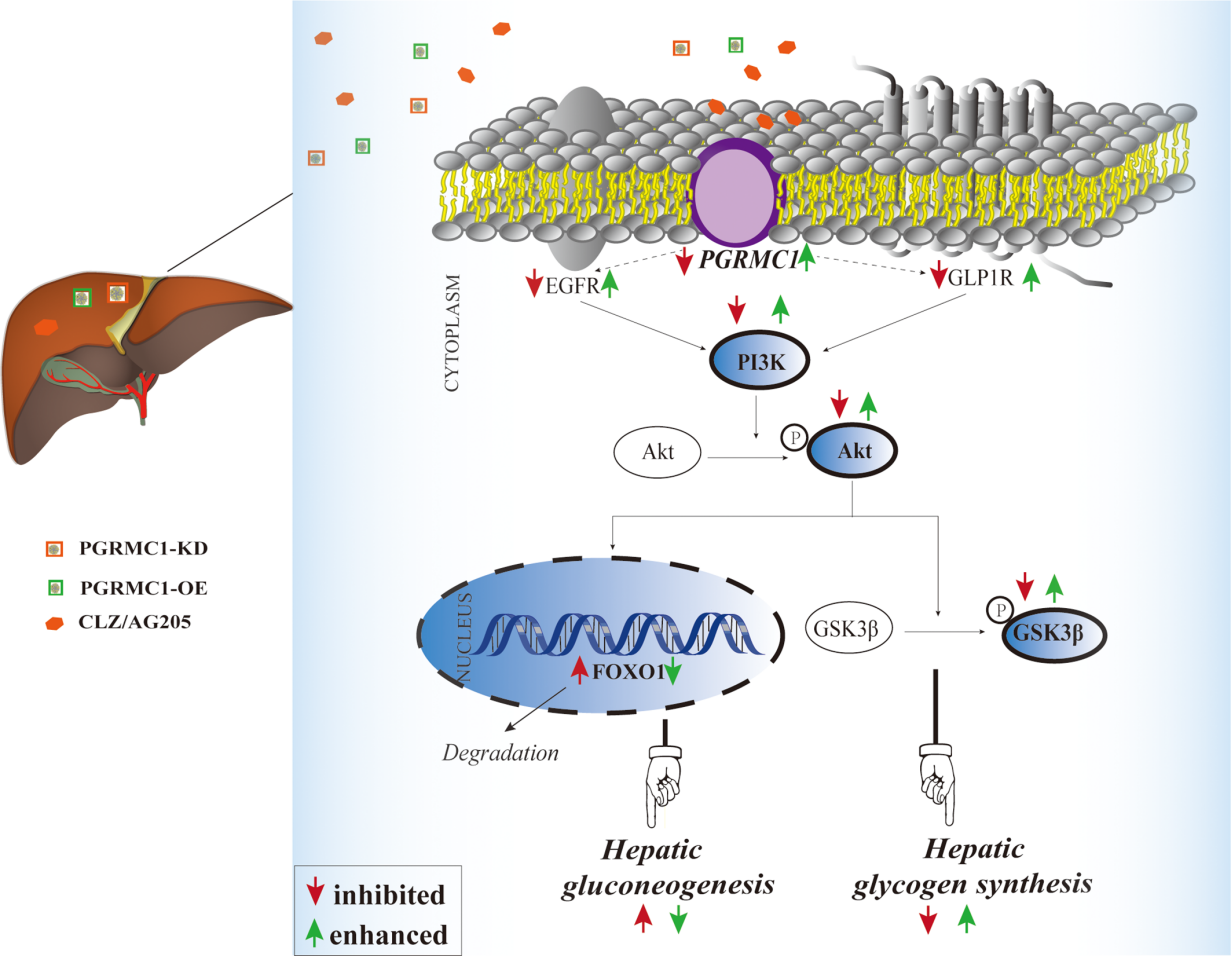
Illustrative model of the mechanism focusing on hepatic PGRMC1 signaling underlying chronic CLZ-induced hepatic glucose disturbances. The add-on PGRMC1-OE can reverse CLZ-induced hepatic glucose disturbances by upregulating the expression of PGRMC1-EGFR/GLP1R-PI3K-Akt-GSK3β accompanied with the downregulation of nuclear FOXO1. CLZ, CLZ; AG205, the specific inhibitor of PGRMC1; PGRMC1-KD, the knockdown of PGRMC1; PGRMC1-OE, the overexpression of PGRMC1; H&E, hematoxylin and eosin staining; PAS, Periodic acid–Schiff staining; PGRMC1, PROG receptor membrane component 1; EGFR, epidermal growth factor receptor; GLP1R, glucagon-like peptide-1 receptor; PI3K, 3-phosphoinositide-dependent kinase-1; Akt, protein kinase B; GSK3β, glycogen synthase kinase-3β; FOXO1, the fork head box protein 1.

The liver plays a crucial role in the maintenance of blood glucose levels by keeping the dynamic balance in gluconeogenesis and glycogen synthesis (27, 28). Increased endogenous glucose production and reduced hepatic glycogen storage contribute to the metabolic abnormalities (28, 29). Our study demonstrated for the first time that PGRMC1 is a key molecule in the hepatic glucose dysregulation induced by CLZ. Primarily, the role of PGRMC1 in regulating EGFR/GLP1R-PI3K-Akt signaling was validated. In rats, knockdown of PGRMC1 mimics, whereas its overexpression moderately alleviates CLZ-induced glucose disturbances. Another important finding is that CLZ increases gluconeogenesis *via* PI3K-Akt-FOXO1 and reduces glycogenesis *via* PI3K-Akt-GSK3β pathway in rat liver. Intriguingly, we found that the PGRMC1-OE effectively reduces fasting blood glucose and glucagon levels, hepatic gluconeogenesis and glucose production and increases hepatic glycogen synthesis. Our study implicated that PGRMC1 may be an emerging target for controlling gluconeogenesis and glycogen synthesis in CLZ-induced hepatic glucose disturbances (as concluded in **Figure 6**).

PGRMC1, also known as 25-Dx, is a member of a multi-protein complex that binds to PROG and other steroids, as well as pharmaceutical compounds (30). As previously reported (15), the downregulation of hepatic PGRMC1 signaling is involved in lipid disturbances occurred following 4-week CLZ treatment in male rats, which is consistent with the inhibitory effects of CLZ on PGRMC1 in our data. Elbein et al. (31) found that PGRMC1 RNA levels decreased in adipose tissue from insulin-resistant subjects, in comparison with insulin-sensitive subjects. Another study (11) revealed diminished PGRMC1 protein levels in high BMI subjects in a small cohort of adipose tissues. From a clinical perspective, the disruption of glucose homeostasis generally keeps accompany with lipid disturbance among the adverse metabolic effects induced by AAPD. Thus, it could be reckoned that PGRMC1 plays a dual role in the regulation of glucose-lipid metabolic disorders. In fact, PGRMC1 has been identified by Selmin et, al. in rat liver (32). However, the function of PGRMC1 in hepatic glucose metabolism is far from fully illustrated.

PGRMC1 has been found to modulate glucose-induced insulin stimulation in beta cells through binding to the liganded GLP1R complex, potentially contributing to glucose homeostasis (13). In general, PGRMC1 is also known to interact with EGFR and further activates intracellular Akt signaling in cancer (19). However, Zhang et, al (13). reported a novel role of PGRMC1 in enhancing GIIS by modulating EGFR-PI3K signaling to potentiate calcium flux and insulin exocytosis. Recently, an antecedent research in our group (24) has proved that PGRMC1 could exert dual effects on EGFR and GLP1R and their downstream PI3K-Akt signaling in rat hippocampus and prefrontal cortex, which provided an explanation for the modulatory effects of PGRMC1 on hepatic EGFR and GLP1R in our study. These inspiring findings above facilitated our exploration the role of PGRMC1 in hepatic glucose disturbances induced by CLZ. In parallel with previous report, our present data also indicated that PGRMC1 protein expression in liver was downregulated following CLZ treatment, so as for the downstream EGFR/GLP1R-PI3K-Akt pathway (**Figures 4**, **5**).

Furthermore, the changes in PGRMC1 expression positively correlated with hepatic glucose disturbances. As illustrated in **Figure 3A**, an increase in fasting plasma glucose levels induced by 4-week CLZ treatment was consistent with a decrease in insulin levels, also suggesting a decrease in insulin secretion. These alternations arising from CLZ treatment are in accordance with a previous report (9) that high dose (20 mg/kg) of CLZ may directly damage the secretory function of β-pancreatic islets, leading to the decrease of insulin secretion. It is well known that insulin regulates gluconeogenesis and glycogen synthesis by activating the PI3K-Akt signal pathway and subsequent GSK3β phosphorylation (33) and FOXO1 inhibition (34, 35). The increase of blood glucose is the result of the decrease of insulin (the decrease of glycogen synthesis) and the increase of glucose production in liver. Conversely, the decreased protein expression involved in the PI3K-Akt pathway may enhance hepatic glycogenolysis and gluconeogenesis (36). Specifically, the downregulation of the PI3K-Akt pathway can induce decreased phosphorylation of GSK3β, thus stimulating the degradation of liver glycogen (16) and further induce FOXO1 translocation into nuclei through reducing phosphorylation of FOXO1 in the cytoplasm, eventually contributing to liver gluconeogenesis (33). In line with the decreased insulin levels as shown in this study, CLZ led to further lower-activated Akt signaling and the reduction of GSK3β phosphorylation, indicating insulin derange. In the present study, we found that CLZ promotes gluconeogenesis *via* inhibition of PI3K-Akt pathway, which is similar to the signal pathway of insulin in the suppression of gluconeogenesis and also partially accounts for the elevation of hepatic glycogen content. Since insulin regulates glucose metabolism *via* PI3K-Akt signaling, the effect of CLZ on insulin secretion and/or on insulin action, at least in part, might explain its capability to induce metabolic disturbances.

As mentioned above, CLZ may regulate hepatic gluconeogenesis and glycogen synthesis by interacting with PGRMC1 thereby inhibiting EGFR/GLP1R PI3k-Akt signaling in the present study. Therefore, we next validated whether the knockdown or overexpression by targeting PGRMC1 exerted the similar or contrary effects on glucose metabolism and its underlying signaling pathway. Using knockdown or overexpression AAV regulating hepatic PGRMC1 levels, we confirmed that rats exhibited disrupted metabolic parameters almost resembling to CLZ’s effects, while the dysregulation was reversed through the overexpression of PGRMC1. However, compared with CLZ group, in the case of no difference in insulin, the blood glucose was significantly reduced by PGRMC1-OE, so it can be seen that CLZ may play a leading role in promoting the production of glucose in the liver. It is interesting that PGRMC1-OE failed to restored the reduced insulin secretion. The potential mechanisms have been elaborated as follows: High doses (20 mg/kg) clozapine may directly damage the secretory function of β-pancreatic islets, leading to the decrease of insulin secretion (9). However, the AAV tool we used for PGRMC1-OE was primarily designed to up-regulate the hepatic PGRMC1 expression thus with less impact in other tissues. However, we are not able to fully rule out this possibility. Evidence has proved that PGRMC1 is also expressed in rat skeletal muscle and progesterone exerts direct action *via* PGRMC1 on glucose metabolism of skeletal muscle (37). Therefore, the PGRMC1-OE used in our study could possibly improve glucose metabolism partially *via* other relevant tissues such as skeletal muscles. AG-205, known as a PGRMC1 inhibitor based on many previous studies (38). We also confirmed that inhibition of PGRMC1 signaling after AG-205 treatment were consistent with results representing PGRMC1 knockdown. Therefore, conditionally upregulating PGRMC1 to promote EGFR/GLP1R-PI3K-Akt signaling may serve as a novel strategy to ameliorate CLZ-induced glucose disturbances.

In addition, hormonal levels might play an important role in CLZ-induced metabolic disturbance especially in glucose dysregulation. It could be hypothesized that CLZ as an inducive factor, lead to hormonal excretion disturbance. As mentioned before, PROG has strong affinity with PGRMC1 among all hormones. Previously, Christine et, al (39) reported that 10 mg/kg CLZ increased the plasma concentrations of PROG in rats. In parallel with disturbances manifested as marked increases of glucose parameters, levels of plasma PROG were also increased in the CLZ-treated rats (**Figure 5D**). Intriguingly, the tendency of increased PROG concentrations was significantly obvious in adrenal gland, followed by the liver. Due to many confounding factors in the blood, plasma PROG concentrations were only elevated in CLZ-treated group. Notably, newly evidence found that exogenous PROG treatment showed strong affinity with PGRMC1 and significantly improved the glucose metabolism of neurons by upregulating the PGRMC1/CREB/GLUT3 and PGRMC1/PPARγ/GLUT4 pathways (40). Combined with our findings, it is likely that the elevated levels of plasma PROG might be viewed as a protective way to ameliorate the glucose metabolic disturbance induced by CLZ. While PROG has been widely demonstrated in the context of ovarian secretion, adrenal synthesis appears to be functionally significant in rodents and may be associated with nonreproductive functions in both males and females (41, 42). Evidence suggests that CLZ acts directly on adrenal enzymes and promotes PROG synthesis, leading to an increase in adrenal PROG production (39). Hitherto, a wide range of preclinical a studies have reported the occurrence of the hormonal excretion disturbance including PROG following AAPD treatments in rats (39, 43, 44). However, it is still not clear about the exact causal relationship. It should be noted that the alternations of PROG concentrations in the tissues and blood are triggered by the action of CLZ, and possibly not directly responsible for the hepatic glucose metabolism disorder.

AAPD treatment is associated with a high risk of significant weight gain in human subjects (45). Notably, CLZ was found to induce less BWG in this study, which did not mimic clinical findings, but was consistent with previous reports in rats (15, 46–48). Similarly, Baptista et al. (48) demonstrated significant reduction in body weight in male rats after 21-day CLZ treatment at a dose similar to our present study (20 mg/kg), which is considered as a result of the sedative effects induced by CLZ. In our experiment, we indeed observed the sedative effects of CLZ on rats, which possibly accounted for the reduced food intake and body weight loss. Evidence indicated that acute CLZ causes a major sedative effect on the rats and loss of motor control and this may have an effect on food intake (8). Albaugh et al. (49) also hypothesized that CLZ may not induce weight gain in rats due to the fact that, relative to olanzapine, it has more marked sedative effects which interfere with eating. Despite well-known sedative/ataxic effects induced by high dose of CLZ (6 and 12 mg/kg), the sedative effects of CLZ at lower doses were not reduced in rats (47). Consistent with our study, impaired glucose tolerance in rats induced by CLZ is associated with increased glucose levels independent of weight gain, indicating an increment in hepatic glucose output (HGO). Increased HGO would in turn increase insulin release, which, however, was not manifested in our study. In addition, the study also indicates that CLZ (10 mg/kg, s.c. for 28 days) can cause derangements in glucose metabolism possibly *via* an increase in glucagon secretion and subsequent stimulation of hepatic glucose production (50). Identical with our study, an elevated tendency of plasma glucagon levels was also observed in CLZ-treated rats without attaining significance, which provided an implication of insulin-glucagon imbalance induced by CLZ. Nevertheless, the present findings suggest significant alternations of glucose metabolic parameters and decreased feeding efficiency induced by CLZ were accompanied with the suppression of hepatic PGRMC1 signaling, which provides the temporal associations between CLZ medication and hepatic glucose metabolic dysregulation.

Based on previous observations, it could be concluded that the 4-week CLZ treatment regulates gluconeogenesis and glycogen synthesis *via* the inhibition of PGRMC1-EGFR/GLP1R thereby regulating the downstream PI3K-Akt signal pathway. The up-regulation of PGRMC1-mediated crosstalk between EGFR and GLP1R signaling pathways may provide a novel strategy for the therapy of disrupted glucose homeostasis induced by CLZ and other circumstances.

## Limitation

On the Day 36 for sacrifice, rats were not administrated with CLZ and the blood were collected from 8 a.m. to 9 a.m. since we aimed to investigate the effects of 4-week CLZ on hepatic glucose metabolism. As evidence reported (51), CLZ indeed caused acute changes in glucose metabolism at 1 and 2 h after administration. In the future, it will be necessary to consider and investigate the acute and chronic effects of CLZ on glucose metabolism simultaneously.

Whether the glucose disturbances we observed herein can be *via* secondary impacts relative to a primary lipid defect is not quite clear. However, as a matter of fact, the disruption of glucose homeostasis usually accompanies with lipid disturbance among the adverse metabolic effects induced by AAPDs. In our previous study (15), we found that the PGRMC1/INSIG-2 signaling played a pivotal part in AAPD-induced lipid disturbances in rat liver. Recently, emerging evidence has also implicated the role of PGRMC1 in glucose homoeostasis, which inspired us to initially figure out the potential mechanism mediated by PGRMC1 in clozapine-induced hepatic glucose disturbances. The interplay between glucose and lipid metabolism is for sure and may contribute to clozapine-induced metabolic side effects. For instance, evidence (9) has shown a strong direct role of clozapine in lipid accumulation and insulin secretion deficiency, further leading to impaired glucose tolerance. Glucose uptake in adipocytes plays a crucial role in accumulation of lipids *via de novo* fatty acid synthesis. Recently, PGRMC1 has been reported to contribute to the acceleration of fatty acid synthesis by upregulating glucose uptake in 3T3L1 cells (52). Further studies are still warranted to disentangle the molecular mechanisms of abnormal lipogenesis from glucose metabolism associated with clozapine actions.

## Data Availability Statement

The original contributions presented in the study are included in the article/**Supplementary Material**. Further inquiries can be directed to the corresponding author.

## Ethics Statement

The animal study was reviewed and approved by The local Ethics Committee of the Second Xiangya Hospital of Central South University (Approval No. 2020008).

## Author Contributions

All authors contributed to and have approved the final manuscript. HC: Conceptualization, Reviewing and Editing. TC: Methodology, Data curation, Writing- Original draft preparation. QC: Methodology, Investigation. BZ, XW, CZ, and SZ: Visualization, Investigation, Validation.

## Funding

This work was supported in part by the grants from Hunan Provincial Natural Science Foundation of China [2021JJ30922], Hunan Provincial Health Commission Research Project [202113010595], Wu Jieping Medical Foundation Funded Special Clinical Research Project [320.6750.2020-04-2], Changsha Municipal Natural Science Foundation [kq2007045], the Fundamental Research Funds for the Central Universities of Central South University [2019zzts1049, 2020zzts884, 2021zzts1073] and New Clinical Medical Technology Project of the Second Xiangya Hospital of Central South University ([2021]94).

## Conflict of Interest

The authors declare that the research was conducted in the absence of any commercial or financial relationships that could be construed as a potential conflict of interest.

## Publisher’s Note

All claims expressed in this article are solely those of the authors and do not necessarily represent those of their affiliated organizations, or those of the publisher, the editors and the reviewers. Any product that may be evaluated in this article, or claim that may be made by its manufacturer, is not guaranteed or endorsed by the publisher.
