# Supplementary material for: Clozapine Induced Disturbances in Hepatic Glucose Metabolism: The Potential Role of PGRMC1 Signaling
Source: Front Endocrinol (Lausanne). 2021 Dec 14;12:727371. doi: 10.3389/fendo.2021.727371 (PMC8712644; doi:10.3389/fendo.2021.727371)
Supplement: Supplementary file 1 [file DataSheet_1.docx]

*Supplementary material*

# Supplementary data

## 1.1 The algorithm used for calculation of AG205 dose

To compare the binding modes of AG205 and CLZ on PGRMC1, a molecular docking analysis was performed. The crystal structure of human PGRMC1 (Protein Data Bank, PDB, http://www.rcsb.org/pdb, code: 4X8Y) was selected as the receptor for molecular analysis. The initial ligand molecule structures (AG205 and CLZ) were retrieved from the NCBI-PubChem Compound Database. The docking simulation was performed with Schrodinger (Schrödinger, LLC, New York, NY, USA), a molecular modeling simulation program that is widely used for the automated docking of ligands and their macromolecular receptors. The conformation corresponding to the lowest energy was selected as the most probable binding conformation.

Molecular docking pattern and score of AG205 and CLZ binding to the sits of SH2 and SH3 of PGRMC1 are obtained and shown in supplementary table 1 and 2. Then, we selected CLZ at 20 mg/kg as a standard for AG205 dose conversion. To mimic the binding effects of CLZ to PGRMC1, the dose of the PGRMC1 specific inhibitor, AG205 was deduced by the molecular docking binding value as illustrated in supplementary table 3.

# Supplementary figures and tables

## Supplementary Figures

## Validation of inhibitory effects of AG205 on hepatic PGRMC1 expression in basal conditions


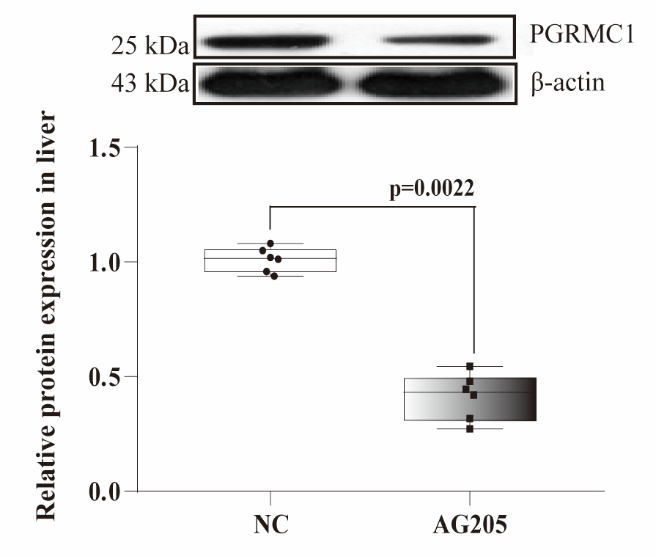


**Supplementary figure 1.** The inhibitory effects of AG205 on hepatic PGRMC1 expression in basal conditions (*n*=6). Mann Whitney U test was used for data analysis.

**The regulatory effect of PGRMC1-KD and PGRMC1-OE in basal conditions**


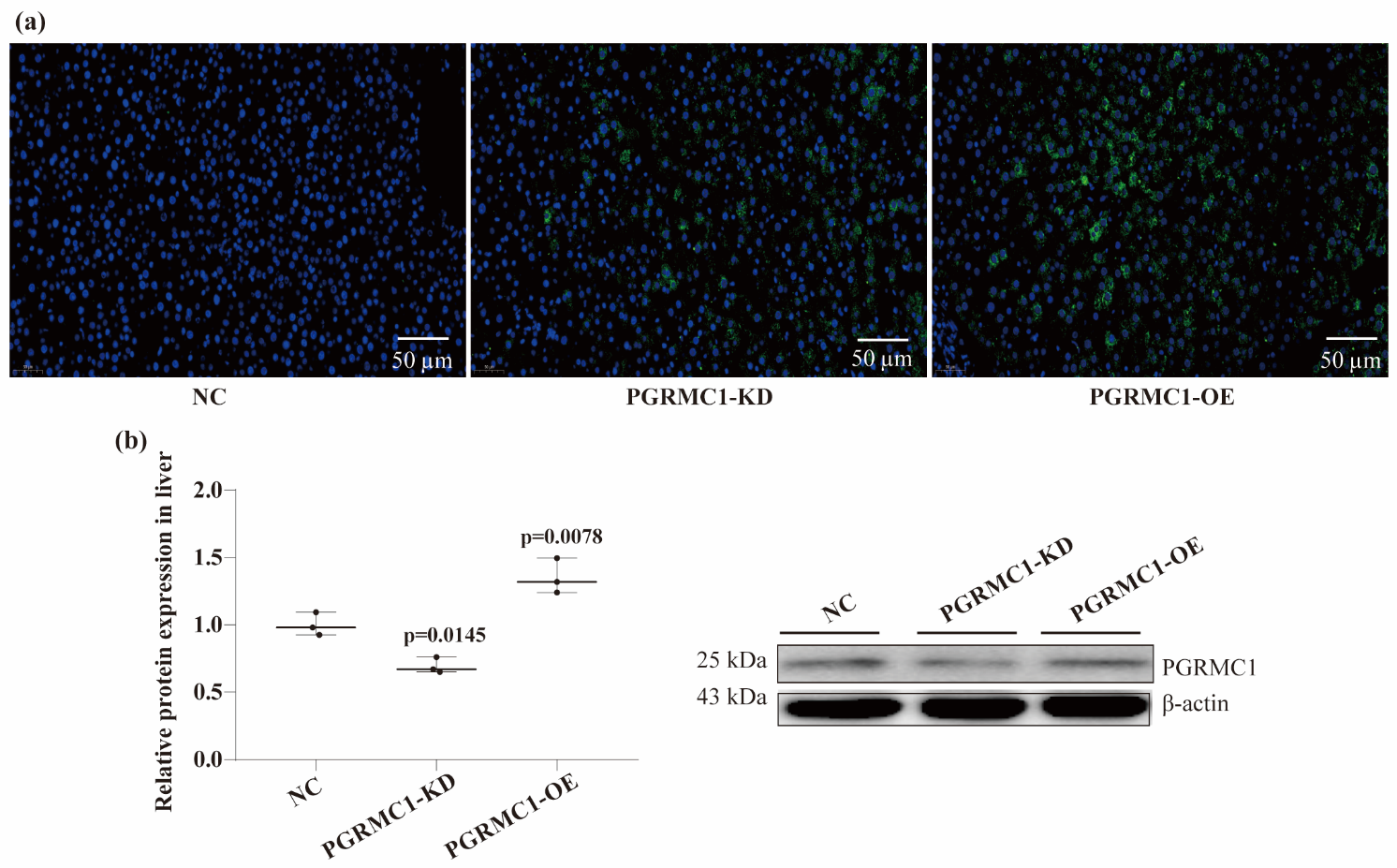


**Supplementary figure 2.** The regulatory effect of PGRMC1-KD and PGRMC1-OE in basal condition. (a) The immunofluorescence of GFP; (b) The PGRMC1 expression in liver.

## Supplementary Tables

**Supplementary Table 1.** Molecular docking pattern and score of ligand molecule binding to SH2 site of PGRMC1.

| Ligand | Docking score | Interaction | Three-dimensional combination mode | Two-dimensional combination mode |
| --- | --- | --- | --- | --- |
| AG205 | -5.695 | Pi-cat: N-Heterocycle---Lys172  H-bond:  carbonyl group--- Lys172 | 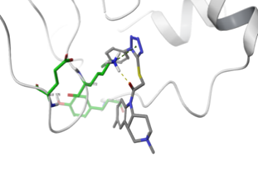 | 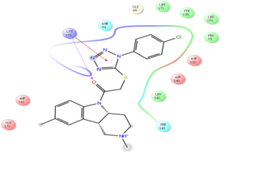 |
| Clozapine | -4.527 | none | 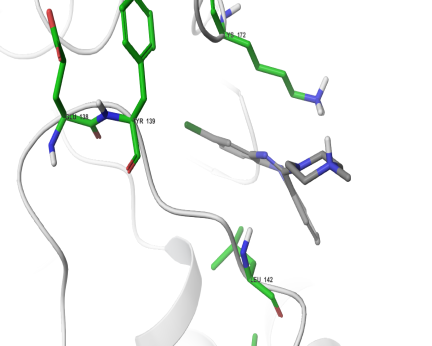 | 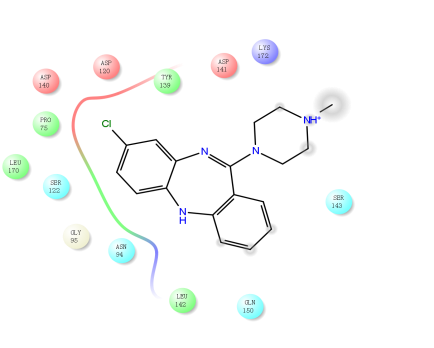 |

**Supplementary Table 2.** Molecular docking pattern and score of ligand molecule binding to SH3 site of PGRMC1.

| Ligand | Docking score | Interaction | Three-dimensional combination mode | Two-dimensional combination mode |
| --- | --- | --- | --- | --- |
| AG205 | -5.794 | Pi-cat:  N-Heterocycle---Gly64 | 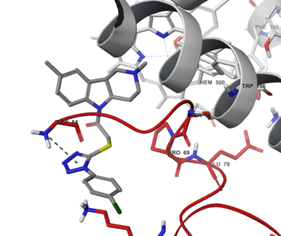 | 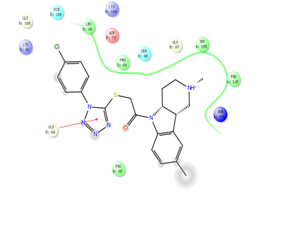 |
| Clozapine | -4.207 | Halogen bond:  Cl atom---Lsy169 | 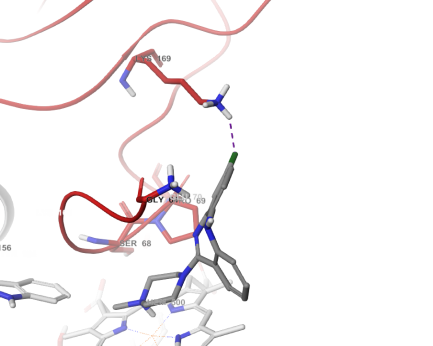 | 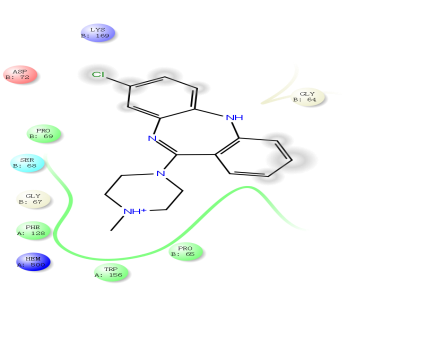 |

**Supplementary Table 3.** Parameters used for dose conversion.

|  | CLZ | AG205 |
| --- | --- | --- |
| Molecular formula | C_18_H_19_ClN_4_ | C_22_H_23_ClN_6_OS |
| Molecular weight | 326.82 | 454.98 |
| Docking score of SH_2_ site  (absolute value) | 4.527 | 5.695 |
| Docking score of SH_3_ site  (absolute value) | 4.207 | 5.794 |
| Total score | 8.734 | 11.489 |
| Molar ratio of CLZ/AG205 | 1:1.315 | |
| Dose | 20 mg/kg | 21.2 mg/kg |

Note: The larger the docking score, the smaller the molar amount needed to achieve the same effect based on the docking theory.

**Supplementary Table 4.** References for clozapine dosage selection.

| **No** | **Title** | **Species** | **Dosage** | **Result** | **DOI** |
| --- | --- | --- | --- | --- | --- |
| 1 | Effects of Long-Term Administration of Clozapine on Body Weight and Food Intake in Rats | Wistar female (weight: 250-280g) and male rats ( 220-250g) | at 0.5, 1, 2.5, 5, 10, or 20 mg/kg (i.p) once daily for 21 days | BW gain during clozapine treatment was not significantly affected in female rats; however, at the dose of 10 and 20 mg/ kg clozapine significantly reduced BW in male rats | 10.1016/0091-3057(93)90084-7 |
| 2 | Clozapine, but not Haloperidol, Increases Brain Concentrations of Neuroactive Steroids in the Rat | Male Sprague–Dawley rats (200–250g) | 10 mg/kg (i.p) once daily for 19 days | 10 mg/kg clozapine also increases the plasma concentrations of progesterone in intact rats | 10.1016/S0893-133X(01)00254-8 |
| 3 | Chronic clozapine treatment in female rats does not induce weight gain or metabolic abnormalities but enhances adiposity: implications for animal models of antipsychotic-induced weight gain | Wistar female rats (about 320g) | low doses (0.25, 0.5 mg/kg i.p, b.i.d for 12 days); middle doses(1, 2, 4 mg/kg b.i.d for 20 days); high doses (6, 12 mg/kg, i.p, b.i.d for 21 days) | clozapine induced a nonsignificant trend toward weight loss over the dose range 0.5–20 mg/kg | 10.1016/j.pnpbp.2007.09.012 |
| 4 | A potential mechanism underlying atypical antipsychotics-induced lipid disturbances | male Sprague–Dawley rats (150-200 g) | 21 mg/kg i.p. per day for 4 weeks | A significant lower percent of weight gain was found in the CLO group after 2- and 28-day treatment than in those of NC groups. | 10.1038/tp.2015.161 |
| 5 | Time-dependent changes and potential mechanisms of glucose-lipid metabolic disorders associated with chronic clozapine or olanzapine treatment in rats | female Sprague-Dawley rats (200–220 g) | 20 mg/kg orally b.i.d for 9 weeks | CLZ lowered fasting insulin levels and impaired glucose tolerance independent of weight gain. | 10.1038/s41598-017-02884-w |

# Construction process and identification of r-PGRMC1 overexpression adeno-associated virus vector

### Sequencing results of r-pgrmc1 overexpression vector

GGATCCGCCACCATGGCTGCCGAGGATGTGGTGGCGACTGGCGCCGACCCCAGCGAGCTGGAGGGCGGCGGGCTGCTTCAAGAGATTTTCACGTCGCCTCTCAACCTGCTGCTCCTTGGCCTCTGCATCTTCCTGCTCTACAAGATCGTTCGCGGGGACCAGCCCGGTGCCAGTGGGGACAACGACGACGACGAGCCGCCCCCGCTGCCTCGCCTCAAGCCGCGTGACTTCACCCCTGCCGAACTAAGGCGATACGATGGAGTCCAGGACCCGCGCATTCTTATGGCCATCAACGGCAAGGTGTTCGACGTGACCAAAGGCCGCAAGTTCTATGGGCCGGAGGGACCATACGGGGTCTTTGCTGGAAGAGATGCATCCAGGGGCCTTGCCACATTTTGCCTGGACAAAGAAGCACTGAAGGATGAGTATGATGACCTTTCTGACCTCACTCCTGCCCAGCAGGAGACCCTGAATGACTGGGACTCTCAGTTCAGTTCACCTTCAAGTACCATCACGTGGGGAAAACTGCTTGAAGGAGCGGAGGAGCCGATTGTGTACTCGGATGATGAAGAACAAAAGATGAGGCTGCTCGGAAGAGTGACTGAAGCAGTCAGTGGAGCATATCTATTTTTGTATTTTGCAAAATCATTTGTAACATTCCAGTCTGTCTTTACAACATGGGGTACC

### Comparison of r-PGRMC1 sequencing results: (the green region is the part matching the target sequence)


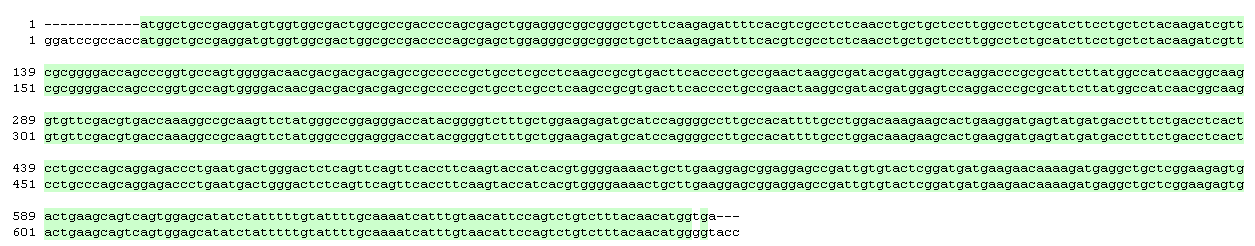


# Construction process and identification of PGRMC1 knockdown adeno-associated virus vector

### 4.1 The sequence information of shRNA designed for selection

The siRNA and shRNA sequences of the control virus vector were designed as follows:

siRNA1 sequence:

GCCGATTGTGTACTCGGATGATGAA

shRNA1 sequence:

| Top strand: AATTCGCCGATTGTGTACTCGGATGATGAATTCAAGAGATTCATCATCCGAGTACACAATCGGCTTTTTTG |
| --- |
| Bottom strand: GATCCAAAAAAGCCGATTGTGTACTCGGATGATGAATCTCTTGAATTCATCATCCGAGTACACAATCGGCG |

siRNA2 sequence:

GAGCCGATTGTGTACTCGGATGATG

shRNA2 sequence:

| Top strand: AATTCGAGCCGATTGTGTACTCGGATGATGTTCAAGAGACATCATCCGAGTACACAATCGGCTCTTTTTTG |
| --- |
| Bottom strand: GATCCAAAAAAGAGCCGATTGTGTACTCGGATGATGTCTCTTGAACATCATCCGAGTACACAATCGGCTCG |

siRNA3 sequence:

GATTGTGTACTCGGATGATGAAGAA

shRNA3 sequence:

| Top strand: AATTCGATTGTGTACTCGGATGATGAAGAATTCAAGAGATTCTTCATCATCCGAGTACACAATCTTTTTTG |
| --- |
| Bottom strand: GATCCAAAAAAGATTGTGTACTCGGATGATGAAGAATCTCTTGAATTCTTCATCATCCGAGTACACAATCG |

### 4.2 The sequencing results

The sequencing results of r-PGRMC1 shRNA1 were as follows:


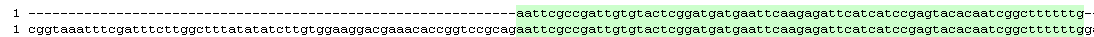


The sequencing results of r-PGRMC1 shRNA2were as follows:


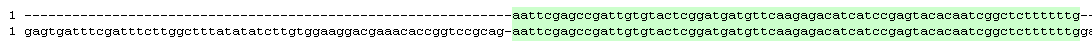


The sequencing results of r-PGRMC1 shRNA3 were as follows:


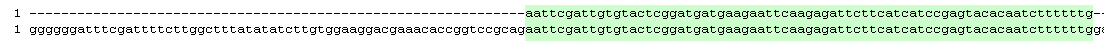


### 4.3 Interference efficiency detected by qPCR

The dissolution curves of GAPDH and PGRMC1 were good, which proved that there was no problem in RNA extraction and qPCR process. PGRMC1 was downregulated in 293T cells, and its shRNA3 interference effect was 71%. Thus, we finalized the shRNA3 sequence for the next step.


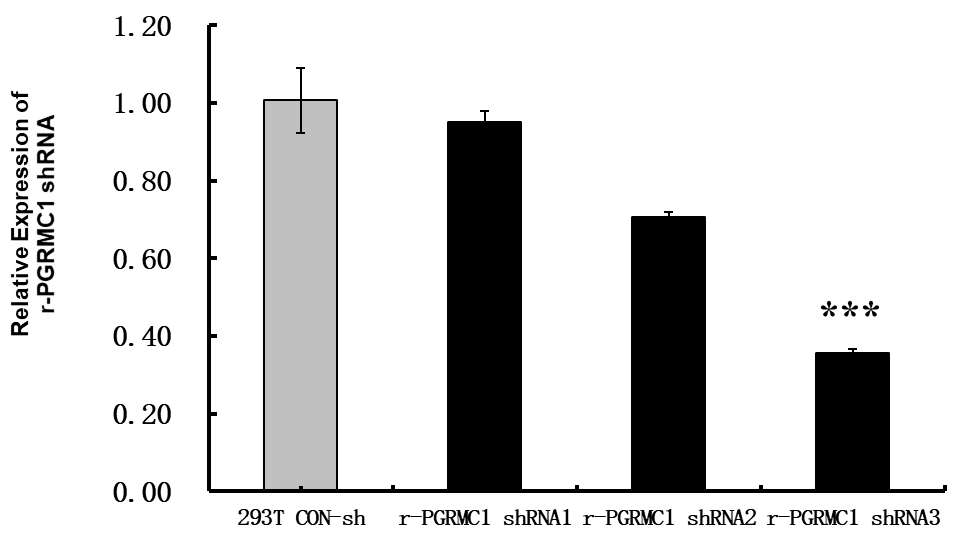


**Supplementary figure 3.** The relative expression of r-PGRMC1 shRNA among the sequences designed in 293T cells.
